# Where is the policy in health policy and systems research agenda?

**DOI:** 10.2471/BLT.15.156281

**Published:** 2015-03-02

**Authors:** Abdul Ghaffar, Lucy Gilson, Göran Tomson, Rik Viergever, John-Arne Røttingen

**Affiliations:** a Alliance for Health Policy and Systems Research, World Health Organization, avenue Appia 20, 1211 Geneva 27, Switzerland.; b School of Public Health and Family Medicine, University of Cape Town, Cape Town, South Africa.; c Department of Learning, Informatics, Management and Ethics (LIME), Karolinska Institutet, Solna, Sweden.; d Radboud Institute for Health Sciences, Radboud University Medical Center, Nijmegen, Netherlands.; e Division of Infectious Disease Control, Norwegian Institute of Public Health: Oslo, Norway.

In the last 15 years there has been considerable growth in the amount of – and funding for – health policy and systems research.^1^ However, research addressing health policy decision-making, across all stages of the policy process, has been relatively neglected.^2^ Here we argue for an increased emphasis on policy in health systems research. We focus specifically on low- and middle-income country contexts where such research has an important role to play in improving health systems and health care delivery.

Health policy and systems research aims to produce new knowledge to improve how societies organize themselves to achieve health goals. Its objectives are to promote the coverage, quality, efficiency and/or equity of the health system,^3^ with the goal of achieving improved health and health equity.^4^

While much health policy and systems research is characterized by its pursuit of better health policies, only a part of it concerns research on policy, i.e. how policies emerge, are formed and are implemented (health policy analysis).^5^ The policy component of health systems research directs attention not only to the formal content and instruments of health policy (the outputs of decision-making) but also to the forces influencing the decision-making: actors, power and politics; institutions, interests and ideas.^6^^,^^7^ The research focus on actors and processes draws from the broad and well-established fields of political science, public administration and organizational science.^8^^–^^10^

Health policies are courses of action and inaction that affect the sets of institutions, organizations, services and funding arrangements of the health system.^11^ Health policy analysis embraces ethnographic and sociologically-informed studies^12^^,^^13^ and considers global influences on health system development.^13^^,^^14^ The analysis supports understanding of the influences on policy agendas and priorities^15^ and how power influences health policy implementation.^16^ It also directs attention to the ways in which health system hardware – the functional and quantifiable pieces – and software – the ideas, values, norms and power that dictate relationships – combine to shape health system functioning.^17^^,^^18^ Health policy analysis also illuminates how past policies, including those directly addressing specific services, programmes or interventions, have unintended consequences on other health system elements or on other health policies. Hence such analyses are part of the complex context of health system development.^17^^,^^19^

From its name, it may appear that the field of health policy and systems research contains two domains: health policy analysis and health systems research. However, the term health policy and systems research indicates that these two areas of work are integrally connected. Health systems respond and adapt to health policies and health policies shape and are shaped by health systems.

To explore the current emphasis being given to health policy analysis within health policy and systems research, we extend an earlier review that examined the research on health policy in low- and middle-income countries.^2^ The review showed that there were only 391 relevant publications between 1994 and 2007.^2^ During this same period there were 35 564 publications for low- and middle-income countries for the whole field of health policy and systems research.^1^ For the present article, we conducted three additional analyses to shed light on the emphasis that is given to health policy analysis within health policy and systems research.

First, we assessed the funding portfolio of the Alliance for Health Policy and Systems Research. The Alliance was established in 1999 in response to a call to establish a “new initiative for research in pursuit of better health policies”.^20^ It is dedicated to developing the health policy and systems research field in its entirety. Yet out of a total of 239 research grants approved by the alliance since its inception until May 2014, only 26 (10.9%) concerned health policy analysis.

**Fig. 1:**
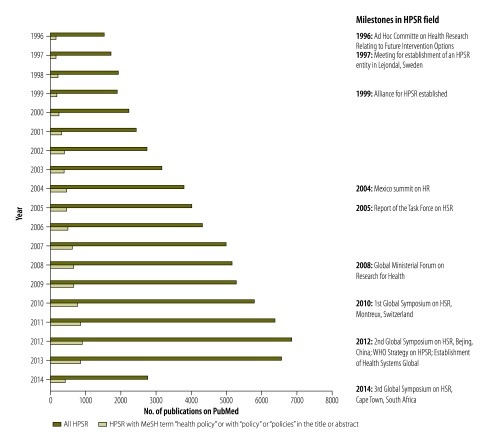
Publications on health policy and systems research and those that contain references to health policy, 1996–2014

HPSR: health policy and systems research; HR: health research; HSR: health systems research; MeSH: medical subject heading; WHO: World Health Organization.

Notes: The number of publications that contain references to health policy is an overestimation of the true number of health policy analysis publications. Many articles are likely to be about research for policy, but not about research on policy.^2^ Compared to other years, numbers for 2014 are an underestimate because of indexing lag. Searches by year were conducted using a search algorithm by Spreckelsen et al.^21^

Second, we conducted a bibliometric analysis examining the volume of health policy analysis and health policy and systems research publications since 1996. We first searched PubMed on 11 February 2015 for health policy and systems research publications, using methods from a recent review on trends in health policy and systems research.^1^ We then assessed how many of these publications were indexed with the medical subject heading (MeSH) term “health policy” or mentioned the word “policy” or “policies” in the title or abstract (Fig. 1). All searches were limited to publications relevant to low- and middle-income countries.

As Fig. 1 shows, only 12.5% (9216/73 712 in the period 1996 to 2014) of all health policy and systems research publications contained references to health policy. However, even this is likely to be a gross overestimation of the true number of publications that specifically investigate policy processes. While these publications may include policy conclusions (resulting from research for policy), only a small proportion is likely to examine the forces shaping policy (representing research on policy).

Finally, we tried to determine whether some parts of the health system may be particularly neglected with respect to research containing references to health policy. We assessed the percentage of publications that were indexed with the MeSH term “health policy” or mentioned the word “policy” or “policies” in the title or abstract for each building block (core functions or dimensions)^22^ of the health system separately for the period 1996 to 2014. This resulted in quite large differences between the six building blocks. Service delivery (19.3%; 4922/25 463 in the period 1996 to 2014), health financing (22.2%; 2666/12 020) and medicines (20.0%; 366/1826) scored relatively highly, with governance in the middle (12.1%; 2208/18 231), and human resources (6.6%; 1608/24 318) and information systems (3.9%; 163/4225) much lower. This suggests that few studies are addressing or generating policy conclusions in the latter three areas.

As with health systems research, there are concerns about the quality of health policy research.^2^^,^^5^ Appropriate theories and analytical methods are often not used, studies frequently lack analytical depth and an explanatory focus and are commonly limited to describing policy problems without developing solutions.^2^^,^^23^ In short, health policy analysis in low- and middle-income countries is still in an early developing phase.

While health systems research has been recognized as an important element for strengthening health systems, there is a relative lack of research on policy, policy processes and their implementation. Given the importance of policy change to health system development, this is a critical gap in the health policy and systems research field. The relative absence of policy analysis funded and published does not align with the objectives of entities in health policy and systems research, major funders of health research and actors in global health in general.

There is also a lack of focus on health policy analysis by key stakeholders, but particularly in a lack of capacity for this type of research in low- and middle-income countries. Therefore, we believe that the global health community needs to enhance its investments in improving capacities in health policy analysis.

The third objective of the Alliance for Health Policy and Systems Research is to facilitate the development of capacity for the generation, dissemination and use of health policy and systems research^24^ and the World Health Organization sees helping research to improve policy-making as one of its core responsibilities.^25^ Therefore, the alliance is launching a new programme of work to strengthen capacities for health policy analysis, especially in low- and middle-income countries.
